# Coming to Grips—How Nurses Deal With Restlessness, Confusion, and Physical Restraints on a Neurological/Neurosurgical Ward

**DOI:** 10.1177/23333936221148816

**Published:** 2023-01-24

**Authors:** Jaco Tresfon, Kirsten Langeveld, Anja H. Brunsveld-Reinders, Jaap Hamming

**Affiliations:** 1 Leiden University Medical Centre, Zuid-Holland, The Netherlands

**Keywords:** ethnography, nursing, patient safety, physical restraints, qualitative, ward care, The Netherlands, Etnografie, Verpleegkunde, Patiëntveiligheid, Vrijheidsbeperkende interventies, Kwalitatief, Verpleegafdeling, Nederland

## Abstract

Physical restraints are viewed as potentially dangerous objects for patient safety. Contemporary efforts mainly focus on preventing bad outcomes in restraint use, while little attention is paid under what circumstances physical restraints are applied harmlessly. The aim of this research was to understand how physical restraints are used by neurology/neurosurgery ward nurses in relation to the protocol. In ethnographic action research, the Functional Resonance Analysis Method (FRAM) was used to map and compare physical restraints as part of daily ward care against the protocol of physical restraints. Comparison between protocol and actual practice revealed that dealing with restlessness and confusion is a collective nursing skill vital in dealing with physical restraints, while the protocol failed to account for these aspects. Supporting and maintaining this skillset throughout this and similar nursing teams can prevent future misguided application physical restraints, offering valuable starting point in managing patient safety for these potentially dangerous objects.

## Dutch Abstract

Vrijheidsbeperkende interventies worden gezien als potentieel gevaarlijk voor patiëntveiligheid. Hedendaagse inspanningen focussen zich voornamelijk op het voorkomen van slechte uitkomsten in het gebruik van vrijheidsbeperkende interventies, terwijl er weinig aandacht is voor de omstandigheden waaronder vrijheidsbeperking schadevrij kan worden toegepast. Het doel van dit onderzoek was begrijpen hoe vrijheidsbeperkende interventies worden ingezet door verpleegkundigen op de verpleegafdelingen neurologie en neurochirurgie in relatie tot het ziekenhuisbrede protocol. Binnen een etnografisch actieonderzoek is de Functional Resonance Analysis Method (FRAM) gebruikt om de dagelijkse inzet van vrijheidsbeperkende interventies en het protocol voor vrijheidsbeperking in kaart te brengen en met elkaar te vergelijken. De vergelijking tussen het protocol en de dagelijkse praktijk liet zien dat omgaan met onrust en verwarring een gezamenlijke vaardigheid is van verpleegkundigen, die van vitaal belang is in het omgaan met en het toepassen van vrijheidsbeperkingen. Het protocol besteed geen aandacht aan deze aspecten. Het ondersteunen en onderhouden van deze vaardigheden binnen het hele verpleegkundige team en vergelijkbare teams, kan toekomstig misplaatst gebruik van vrijheidsbeperkende interventies voorkomen. Dit biedt een waardevol startpunt voor het managen van vrijheidsbeperkingen als potentieel gevaarlijke interventies binnen de verpleegkundige zorg.

## Introduction

To this day, the usage of physical restraints is still a widespread practice in nursing. Physical restraints are controversial, as these are seen as limiting the patients’ freedom and are potentially dangerous objects for patient safety (Evans et al., 2003; Kontio et al., 2010). This tension shapes a general tendency toward viewing physical restraints as ultimately unwanted. Still, nuances can be made to the practice and debates remain about the aptness of the intervention (Funk & Bold, 2020; Sokol, 2010). Furthermore, prevalence numbers vary between countries and healthcare settings making it hard to seize the full magnitude of this issue (Ambrosi et al., 2021; Krüger et al., 2013; Lee et al., 2021; Thomann et al., 2021).

The use of physical restraints has been associated with several physical and psychosocial forms of harm (Rakhmatullina et al., 2013). Relatively mild complications are noted in the prevalence of pressure ulcers, joint injuries or increased risk for delirium. In more severe cases, strangulation is reported to be related to restraint use. Psychosocial factors affecting the patients are for example feelings of distress and dehumanization, while nurses report feelings of fear, guilt, and conflict when applying restraints. Furthermore, where restraints are used, the individual caregiver can be endangered by patients themselves (Renwick et al., 2016).

There are important ethical considerations in balancing between the proportionality of the used intervention and retaining patient autonomy (Gastmans & Milisen, 2006). Insights into the decision making of nurses lying behind restraint use show an ethical dilemma of a continuous trade-off between patient safety and patient autonomy (Goethals et al., 2012; Kontio et al., 2010; Marangos-Frost & Wells, 2000). A nurses’ decision which restraint is appropriate in a given situation is a balancing act that continuously addresses the patients situation rather than following clear-cut rules (Goethals et al., 2012). Such concerns reflect the difficult considerations accompanying the use of restraints and their implications for nursing guidelines.

Since physical restraints are a dangerous intervention accompanied by difficult considerations, guidelines and protocols should support nurses and provide aid how these instruments can be dealt with sensibly and safely. Contemporary research and policy efforts acknowledging the hazardous nature of restraints on the other hand treat physical restraints as an object ideally omitted from daily care (Fariña-López et al., 2018; Krüger et al., 2013; Via-Clavero et al., 2019; World Health, 2019). Studies aiming at reducing restraints over the last 30 years however have shown limited results (Abraham et al., 2020). In the Netherlands, a national guideline for restraint use in hospitals was developed so that restraints are less used or applied diligently (V&VN, 2013). Since hospitals used their own protocol or guideline, this guideline was developed to offer an unambiguous tool for general and academic hospitals to use. Its central tenet, “Restriction of freedom? No, unless. . .”, likewise suggests that restraints are viewed as an ultimately unwanted practice. While this might be appropriate from an ethical and judicial perspective, nurses still have to consider restraints in trading-off patient safety and autonomy. This raises the question how Dutch nurses are indeed supported by this document and the translation into a hospital protocol in everyday activities, and how this can be effectively analyzed.

With a critical intervention such as physical restraint use, the extent to which protocol in adherence plays a part is important. In recent years, patient safety studies have used two viewpoints toward work as a means to understand how designed and prescribed work processes (e.g., audits, quality improvement projects, indicators, guidelines, protocols) on the one hand, support activities as they are actually done in practice on the other hand (Anderson et al., 2020; Braithwaite et al., 2015, 2016; Furniss et al., 2019; Pedersen & Mesman, 2021). The distinction goes beyond compliance, as it acknowledges that nurses have to adapt to changes and surprises to deliver patient centered care. This suggests that a gap might exist between prescribed activities and actual practice, but this gap could also offer directions for further improving patient safety (O’Keeffe et al., 2015; Vos et al., 2020). Such approaches (e.g., Safety-II, Resilient Health Care), can offer a fresh perspective on improving patient safety, as these don’t start by examining how unwanted outcomes can be prevented in the future, but rather try to thoroughly understand what abilities professionals already possess in creating safety (Iflaifel et al., 2020; Verhagen et al., 2022; Waring & Rowley, 2011). This might also be the case for applying physical restraints in nursing. Analyzing how deviations from protocols and adaptations contribute to safety on a daily basis, could show how further use of physical restraints can be reduced.

Restraints are typically used in nursing homes or in the context of mental healthcare. Aside from the much studied Intensive Care Unit setting, often neglected is physical restraint use on other acute hospital wards (Thomann et al., 2021; Xyrichis et al., 2018). On neurological wards, patients can also be prone to confusion and at-risk behavior due to their clinical condition (Gilbert & Counsell, 1999). While this heightens the chance of physical restraint use by ward nurses, it also forms a suitable context to study physical restraint use as part of everyday care on hospital wards.

This papers aims to illustrate how reviewing work descriptions against actual practice can be used to improve physical restraint use and patient safety in nursing. To appreciate and contextualize the daily activities on the ward, the role of prescribed practice standards is included as a point of reference. This allows for a comparison and helps to understand what aspects of daily physical restraint use are presently supported by protocols. The objective of this study is to perform an extensive collaborative study to understand how the guideline and protocol of restraint use relate to and support actual nursing practice on a neurological/neurosurgical ward, and to what extent nursing practice already incorporates safe aspects of dealing with physical restraints.

## Methods

### Theoretical Concepts and Analytical Approach

#### Safety-II and resilient health care

In comparing to what degree the guideline and protocol of physical restraint use indeed help to deal with restraints safely, we draw on the theoretical concepts of Safety-II and its application field in healthcare, Resilient Health Care (Braithwaite et al., 2015; Hollnagel, 2018; Hollnagel, Braithwaite et al., 2013; Iflaifel et al., 2020). In short, both schools of thought originate from the safety science research community, and have come forth from an unease with conventional safety management approaches being deployed within an increasingly complex world (Dekker, 2019; Smaggus, 2019). Both Safety-II and Resilient Health Care see the capabilities of health care workers to deliver care under expected and unexpected situations as the driving force behind high quality care (Wiig et al., 2020). This capability, called resilience, is believed to cause both wanted outcomes (i.e., safe ordinary care, high quality care) and unwanted outcomes (i.e., adverse events). Rather than a sole prevention of unwanted outcomes, Safety-II and Resilient Health Care advocate for a well-founded appreciation of people’s everyday problem solving and coordination skills in addition to learning from error and mischief. The perspective of Safety-II and Resilient Health Care allows you to look at existing practices in a different way, so that one can better understand how both negative and positive outcomes come about. Consequently, it is important to thoroughly understand the perspectives and everyday work context of healthcare professionals when studying safety hazards as part of daily activities in large health systems (Berg et al., 2018; Leslie et al., 2014).

#### Functional resonance analysis method

A popular method to describe and achieve greater understanding of all activities and outcomes in (patient) safety can be found in the Functional Resonance Analysis Method (FRAM) (Hollnagel, 2012; Patriarca et al., 2020). FRAM is a modeling method which can be used as a tool to model, give insight and learn from activities, instances or an entire process in complex organizations, with the aim of understanding of- and learning from how performance outcomes arise. FRAM focusses on activities and interrelations within a process, helping to translate and interpret observed actions and events. FRAM can help structure observations and data from interviews in healthcare processes through development of a model. Coding fieldnotes for actions and events related to the healthcare process under study is a first step. Codes are categorized for related activities, and unique actions and events are then listed and transformed into verbs. The activities described by the verbs form the basis of the FRAM. Coupling these activities and clarifying the nature of the relationships is the next step. This shows the interrelations between the activities within the process. An activity can be an input (I) of another activity, or reversely an output (O). Other relationships in FRAM between activities are preconditions (P), resources (R), controlling activities (C) or time dependent activities (T). FRAM models are easily re-shapeable and thus ideal for iterative research design. FRAM as a method allows for the construction of several different pathways and interdependence in a process. As such, it forms an optimal method to map a process in which different perspectives and working methods are present. An insightful illustration and explanation of using FRAM in healthcare can be found in Clay-Williams et al. (2015).

FRAM has earlier been used to understand how activities and adaptions unfold in ward care (Kaya et al., 2019; Raben et al., 2018) and hospital-to-home transitions (Salehi et al., 2021), or for highlighting differences between actual and prescribed or planned working practices in clinical settings (Clay-Williams et al., 2015; Damen et al., 2021; Schutijser et al., 2019). These studies illustrate how adaptations arise and what reasons and consequences are associated with deviations from guidelines. Giving notice to such applications, FRAM could be a feasible approach toward understanding everyday use of physical restraints.

### Study Design

#### Background

The present study was part of a larger ethnographic action research project aiming to (1) enhance ownership over healthcare processes and quality improvements among ward nurses through action research, and (2) understand how care quality assessment instruments and prescribed work instructions on the ward (i.e., work-as-imagined) relate to everyday activities on the ward from the perspectives of the ward nurses (i.e., work-as-done). For this second aim, we performed an action research study in which daily activities and the protocol of physical restraints were compared and aligned from the perspectives of ward nurses. In this paper, we report on the findings resulting from the comparison between practice and the protocol. A more elaborate report of the entire realignment efforts on the ward can be found in Tresfon et al. (2022).

We used ethnographic methods for over a 2-year period on the ward to gain a deeper understanding of the emic perspective of the ward nurses as this approach resonates particular well with the goal of studying local cultures and practices. Hospital ethnographies following the professionals’ perspective are increasingly popular methods in studying quality and safety in-depth within hospitals (Catchpole et al., 2017; Dixon-Woods, 2003; Leslie et al., 2014; van der Geest & Finkler, 2004). Following the literature on resilient healthcare (Hollnagel, Braithwaite et al., 2013), its popular FRAM method (Hollnagel, 2012; Patriarca et al., 2020) and foundations rooted in viewing healthcare through a lens of complexity science (Braithwaite, 2018; Braithwaite et al., 2015; Pedersen, 2016; Wiig et al., 2020), attaining a comprehensive understanding of the motivations and views of local actors is a fundamental aspect of appreciating how and why phenomena and outcomes emerge in complex healthcare processes. By placing multiple perspectives on common working methods of the nurses at the center of our research, our ethnographic approach is rooted in an interpretivist epistemological stance.

#### Setting

Our inquiry took place on a combined neurological and neurosurgical ward of a tertiary hospital in the Netherlands consisting of 38 patient beds and a mixed nursing workforce of approximately 80 nurses. During the initial exploration phase of the research project between February 2020 and October 2020, physical restraints were noted as a topic of particular interest for both management and the nurses on the ward, since neurological patients are due to their illness more prone to confusion and endangering self and others. After exploring the actual restraint use on the ward in relation to the protocol, an action research project was started from October 2020 to June 2021 to see how research findings could be embedded in practice. The present study reports on the initial findings of this ethnographic action research comparing practice and protocol concerning physical restraints during the exploration phase.

#### Participants

Through purposive sampling, 15 nurses, one nursing manager, one physician assistant, one clinical manager and one quality and safety advisor were included in the FRAM model interviews and member checks. The main inclusion criteria for the nurses was differential years of experience on the ward (<1 year to 20+ years). Managers and advisors were included to expand the knowledge about the role of formal written guidance. This offered a diverse pallet of perspectives and experiences with daily restraint use and the protocol. Nurses eligible for inclusion were recruited through the wards’ nurse manager or invitation while observing on the ward.

### Iterative Research Process

We used an explorative iterative approach drawing on ethnographic methodology to study the perspectives and working practices of the nurses in their daily work lives as a basis for a comparison with written guidelines, protocols and other work instructions. For this purpose we developed two FRAM models. The first model was primary interested in the actual use of physical restraints on the neurological/neurosurgical ward. The second focused on all work prescriptions and descriptions concerning restraint use by nurses within the ward and hospital. Comparison between both models served to comprehend how formal written guidance related to and supported normal restraint use on the ward. The iterative development of the two FRAM models guided the concurrent data collection and analysis.

#### Data collection

Methods for data collection included participant observations, single and group interviews, and an extensive member check. Also, documents on the use of restraints were consulted such as the national nursing guideline, the hospital wide protocol and other affiliated work documents (e.g., various step-by-step plans for dealing with at-risk behavior such as delirious or aggressive patients, related protocols, manufacturer and hospital user manuals of the physical restraints themselves).

Observational data were collected through 10 participant observations and in-field note taking, which were afterward elaborated in fieldnotes. Initially, observations were done by hanging around on the ward and visiting team meetings. Subsequent participant observations were done by shadowing nurses and physicians on the ward during daytime shifts, wearing a nursing suit or doctor’s coat. Based on these observations, an initial FRAM model of restraint use was made.

Interview data were collected during two separate interviews with a nursing expert from the ward and a senior quality and safety advisor from the hospital, and a group interview with three ward nurses of varying work experience on the ward (less than 1 year, 7 years and 20 years). Interviews were carried out by two researchers (JT and DvV), whom presented the latest version of the FRAM models on A3-paper to the participants during the interviews. The FRAM models provided the structure and topics for the interviews. Interview questions were guided by discussing contemporary working practices, and to what extent guidelines, protocols and other work descriptions were similar, precise and helpful in respect to everyday activities. Interview data were collected by note-taking of the researchers during the interviews, comments and annotations made to the FRAM models by the participants, and discussions between the researchers after the interviews. A complementary research diary was kept to structure the iterative process of FRAM model development and used during analysis.

#### Data analysis

The observations and interviews formed main input for the development of the FRAM model of actual restraint use. The hospital wide protocol formed the basis for the FRAM model of prescribed restraint use. FRAM models were built using the FRAM Model Visualizer software tool. During each interview, the models were discussed and fine-tuned based on the feedback from the participants. After the interviews, two researchers (JT and DvV) discussed the implications of the feedback further and incorporated this in the FRAM models accordingly. Following the aim of the study, the FRAM models were refined with every interview until the difficulties met in practice, the use and value of the protocol and important aspects of restraint application (missing in the protocol) became sufficiently clear. FRAM in this study was thus used as an iterative method propelling deeper understanding of the ward nurses’ activities and perspectives on the application of restraints on the ward, as well as the added value of the protocol.

Comparison of the two FRAM models resulted in the construction of four main themes important for distinguishing between normal daily restraint use and formal written guidance. Since theoretical nor thematic saturation were the aim of our study and more generally might be hard to achieve (Braun & Clarke, 2021; Thorne, 2020; Varpio et al., 2017), we performed a member check after initial analysis to enhance trustworthiness of the interpretations made on basis of the FRAM models. While our iterative approach allowed us to deeply explore and continually reinterpret the use of restraints by the nurses and the value of the protocol (Varpio et al., 2017), we still wanted to ensure that our interpretations and recommendations based on the data analysis sufficiently and understandably addressed the vital aspects of restraints application met in practice. To do so, the member check was done by summarizing the findings in a three-page description of contemporary working practices of restraint use on the ward. The description was discussed with 11 nurses of varying experience, clinical and nursing management and a physician assistant on the ward and revised iteratively. After minor revisions, a final FRAM model based on the new description of working methods was made for interpretation. The identified themes were further triangulated by data from observations and interviews.

#### Triangulation

Adjacent to the above research efforts guiding the FRAM iterations, field notes from 12 additional participant observations and transcripts from 18 semi-structured interviews with the ward nurses were included in the analysis for the sake of triangulation. The participants observations were performed during day, evening, and nighttime shifts (8 hours per shift) by shadowing nurses, physicians, medical secretaries and nutritional assistants. Data was again collected by in-field note taking and afterward elaborated on in field notes. The semi-structured interviews took on average 45 minutes each and focused on six topics (psychological safety, job satisfaction, workload, autonomy, locus of control, and prevention of incidents). The interviews were recorded and transcribed verbatim. Whereas the observations were performed throughout the FRAM-interviews and member check periods, the semi-structured interviews were held after the FRAM model development was finished, while the member check still took place.

### Reflexive Account

With no background in medicine or nursing but a master degree in cognitive psychology and a theoretical background in general safety literature, researcher JT had little assumptions or prior experiences with ward care. During the data collection period, JT got increasingly acquainted with the customs and relational dynamics on the ward as a consequence of preparing for the action research project. While the observations and FRAM interviews were held as an ethnographer on the boundaries of the group, during the member check JT was an active action researcher on the ward.

### Ethics

The study was reviewed and found of no concern by the Medical Ethical Board Leiden Den Haag Delft [N20.019/ML/ml]. Consequently, the need for written informed consent was not applicable. All nurses on the ward were informed repeatedly by management before and during the data collection period on the ward through email, newsletters, and during team-meetings. Consent was acquired verbally throughout the observation period and before interviews. Retrieved data was ensured to be not relatable to specific individuals.

## Results

While discussing the FRAM and the subsequent description of working methods, four themes were identified: (1) Actual process versus the protocol, (2) dealing with restlessness and confusion, (3) collaboration and shared experience, (4) and expect the unexpected. Whereas the first theme describes how the protocol related to actual practices and viewpoints on the ward, the three other themes set out to describe three important aspects of applying restraints in practice which were missing in the protocol.

### Actual Process Versus the Protocol

The FRAM analysis revealed that the general process of applying restraints in practice consists of several important actions starting well before the restraints are actually considered and applied (see Supplemental Files). Starting with the suspicion that restlessness is developing, the ward nurses subsequently engage in a multitude of tactics in order to distract or comfort the patient in times of restlessness or confusion, preventing subsequent at-risk behaviors from becoming unmanageable and physical restraints from being necessary. When the act of restraining is considered, the nurses mutually discuss the appropriate restraint type, collaborate to take the right course of action at that time, and aid each other while applying the restraint. In doing so, the nurses hold on to three principles when having to deal with restlessness and/or confusion, and so the possibility of a physical restraints being necessary: Firstly, “Keep the freedom of the patient as great as possible,” secondly “Deliberate with colleagues” and thirdly, “Consult the physician and legal family representatives (at an appropriate time).”

**Figure 1.**
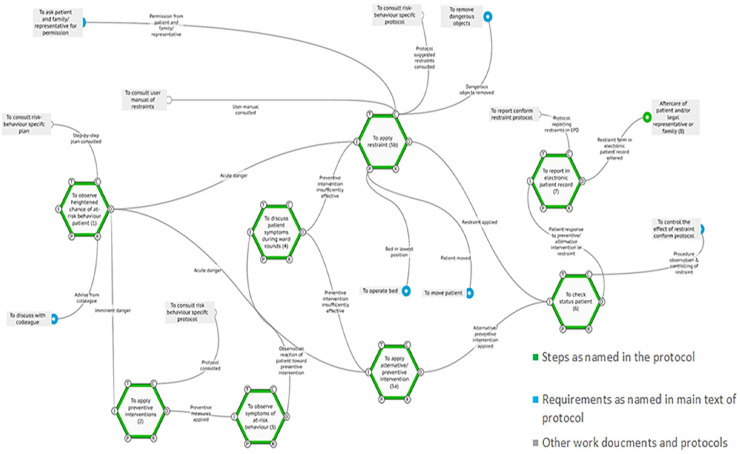
FRAM of protocol. The FRAM model of prescribed restraint use according to the hospitals’ protocol. Reviewing the protocol showed strong similarities with the national guidelines, which use the same steps as named in the protocol. *Source*. Adopted from Tresfon et al. (2022).

Being allowed to apply restraints on the ward all together was regulated by two forms of education: a hospital-wide mandatory e-learning, and a practical teaching on the ward concerning how to apply restraints safely. The protocol however was seldom used. In fact, discussing the FRAM of the hospital protocols and affiliated work documents, these were reported to be of limited usefulness. The documents were only helpful in cases of doubt and consulted when there was enough time to do so, but often commented on as outdated, inconvenient in use or lacking clarity. The hospital protocol contained references to the national guideline, and the FRAM analysis showed that the protocol was almost entirely similar the guideline in the order of prescribed steps to be taken (Figure 1). The hospital protocol further contained reference to other protocols and work instructions as means for further instruction. In the protocol, no attention was paid to signaling restlessness or the importance of collaboration, and the use of alternative measures was only sparsely referred to and seldom practically described. In comparison to the guideline and protocol both stating physical restraints as a “no, unless.” activity, the principles of the nurses in practice reshaped and reemphasized the fundament of the guideline and protocol. This way, the nurses can follow the spirit of the guideline while maintaining a practical approach toward dealing with restless and confused patients.

Remarkably, the nurses had a different interpretation what could be considered a physical restraint in comparison to the protocol. Following the national guideline, the protocol largely specifies and categorizes all restraints based on restriction of freedom, ranging from relatively mild interventions (camera surveillance) to the most heavy and intrusive (abdominal, ankle, and wrists bands). The mild categorizations distinguish between physical (e.g., care mittens), non-physical (e.g., posey bed) and electronic restraints (e.g., acoustic fall detector), whereas the heavy categorizations refer to the abdominal, ankle, and wrist band. For all categorizations, the protocols describes in detail all steps that must be taken before a physical restraint is used, but practice shows that this mainly applies to restraints that clearly restrict a patient’s freedom: care mittens, a posey bed and abdominal, ankle, and wrists bands. Going through all the steps in the protocol for lighter interventions seems nonsensical to the nurses. If a patient is put in a wheelchair for an hour because it calms him or her down, the nurses do not feel compelled to put this in a separate registration form or to “annoy” the doctor with it. Bed rails did not seem to be seen as a physical restraint at all, but are used in consultation with the patient when used.

### Dealing With Restlessness and Confusion

The process of applying physical restraints on the ward was reported to be much more extensive than the act of restraining confused patients. In fact, the nurses initially try to prevent confusion from occurring by monitoring and mitigating signs of restlessness, a clinical nursing indicator on the ward that a patient can become confused and show at-risk behavior. Restlessness is initially suspected based on patient history from the nursing anamnesis or clinical handover, but signs can also be observed during shifts. When a patient becomes restless, for instance when fidgeting at the bed sheets is noted, this is an indicator for the nurse to look for a deeper cause of the patients’ behavior. Looking for such causes is important, since dealing with these causes can take the restlessness away and prevent confusion and at-risk behavior to arise. The nurses report several causes that can be attributed to restlessness (Table 1), but not always a direct cause is found. For some patients it can be also considered to administer calming medicine, so called “escape medicine,” but due through the disturbance on the neurological observations for both the nurse and physician, this is often uncalled for.

**Table 1.** Overview of Preventive and Alternative Measures Used by the Nurses to Prevent and Mitigate Restlessness and Confusion, as Well as Creative Solutions Used as Markers for at Risk Behavior.

Preventive measures	Alternative measures	Alternative measures	Creative solutions
*Measures aimed at finding and mitigating the cause of restlessness.*	*Measures aimed at calming the patient down in a low-stimulus environment.*	*Measures aimed at distracting the patient from the underlying suffering.*	*Solutions used for signaling and anticipating at-risk behavior.*
Full bladder	Placing the patient in a single room.	Making small talk with the patient.	Placing a bedpan lid on the doorhandle.
Constipation	Rooming-in of family.	Handing the patient a magazine.	Stickering the wards’ name on the back of wandering patients.
Pain	Placing the patient closer to the nursing station	Letting the patient watch TV or make a puzzle.	Closing the doors of the ward, opened only by light switch button next to the door.
Shortness of breath		Planning a daily schedule for the patient.	Informing security of potential wandering patient
Delirium		Giving the patient household chores (e.g., folding towels, tidy up the bedside table).	
		Bringing the patient to the wards’ living room.	
		Seating the patient in a wheelchair in the hallway.	

**Figure 2.**
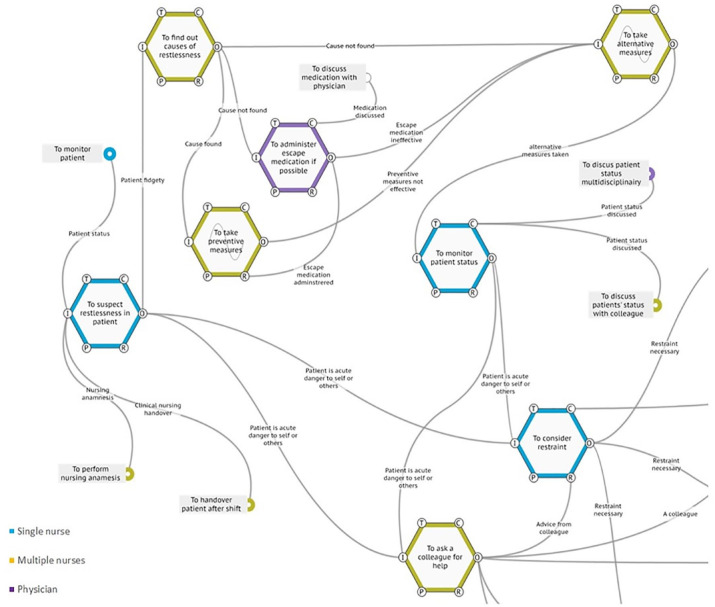
FRAM of actual practice, early process. Dealing with restlessness and confusion through a multitude of mitigating and diversion tactics was seen as of vital importance early on in the process of applying restraints. The process of restraints is predominantly a nursing intervention, in which nurses cooperate to find the appropriate solution to the case at hand. In the FRAM, this is illustrated by differentiating between single nurse (blue) and cooperation between nurses (yellow). When a physician was consulted, the function is purple. The light gray waves in two function mark the variability in knowledge about preventive and alternative measures.

When preventive measures do not suffice, the nurses engage in alternative measures to calm down the patient. These tactics are aimed at either placing the patient in a low-stimulus environment or distracting the patient from the underlying suffering, depending how the patient reacts to the taken measures (Figure 2). The nurses use these measures or “tricks” to prevent subsequent at-risk behaviors from becoming unmanageable and restraint use from being necessary.

Commonly known at risk behaviors which could arise on the ward were risk of falling, delirious behavior, slipping out of bed or chair, physical aggression, a tendency to remove medical material or (night) wandering. While some of these behaviors readily made the patient a danger to the self or others, in others an ad-hoc risk assessment is needed to weigh how much the nurse trusts the patient for the time being while caring for other patients. When interviewing one of the nurses, she explained how she anticipates on confusion in the light of preventing incidents from happing:

*For example, falling. That’s a really big thing here. It is not the case that we will order a tent bed [posey bed] for everyone by default. But you tell them [patients] emphatically that they have to call when they have to go to the toilet. Or, when you hear that there will be an admission, “yes he is familiar with dementia, he is a bit confused,” you put them a little closer to the counter [nursing station]. Also for the night shift, so you can see them [patients], when they go to the toilet. (Interview 6)*

For some confused patients on the ward delivering care becomes increasing difficult when a patient has a lack of insight in their own clinical condition, occasionally ignoring for instance palsy limbs or cognitive-visual impairments.

### Collaboration and Shared Experience

**Figure 3.**
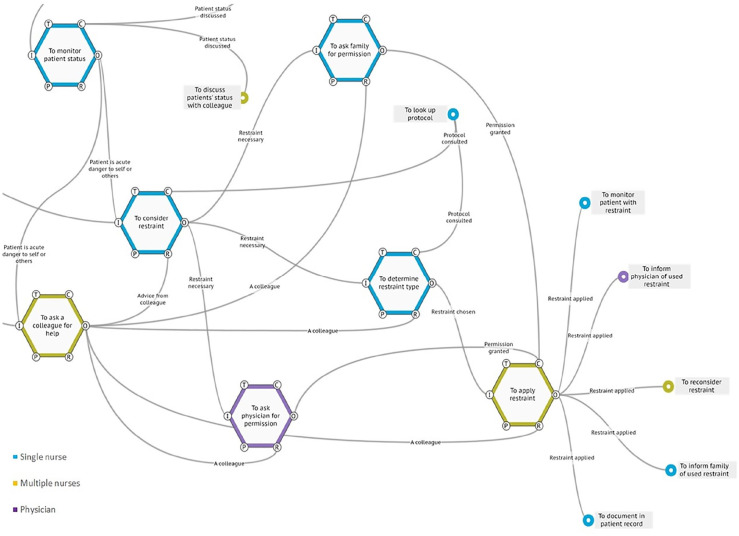
FRAM of actual practice, considering application. Recognizing the current situation, weighing the appropriate action to take and the application of physical restraints was reported to be a shared act between the nurses, in which they rely on both each other’s previous experience with the patient, experience with similar patients and hands-on support.

Recognizing which situation can develop and how to deal with it appropriately is a matter of experience and collaboration between the ward nurses. This is true for the dealing with restlessness and confusion, as well as deciding on the appropriate physical restraint to use and subsequent safe application (Figure 3). Both during and after restraint application, cooperation and making use of each other’s prior experience with the patient and physical restrains in general was of vital importance for the nurses. During the group interview this case was repeatedly stressed, explaining that “applying physical restraints isn’t something you solely just do.” Aside from being practically almost impossible, it can be very dangerous when the patient literally outweighs the nurse.

Monitoring the implemented alternative measures is a continuous act in which the nurses notify each other and discuss the implemented interventions among themselves, seeking to weigh the situation from different knowledge sources. While this is true for reporting patient experiences between shifts, also advice based on experience with comparable situations plays a large role. The more experienced nurses were said to show a better understanding of how to deal with restlessness and confusion, being aware of a larger amount of preventive and alternative interventions and signs that the intervention was the appropriate route to take compared to less experienced nurses. During the daily ward rounds the current situation of the patient is discussed with the physician, or ad hoc in case the patients’ situation grows worse. During observations it was noted that some alternative measures were embedded in the ongoing activities of the ward nurses. For example, a coordinating nurse checks with her colleagues which patients can be admitted to the wards’ living room:

*Back on the ward, the Stip [coordinating nurse] goes back to the counter, the usual lookout location of the Stip. She walks to the pharmacy behind the counter and asks one of the nurses present there if she has completed the list for the living room visits. Living room visits have been a thing for a few months now because volunteers on the ward sit in the living room with patients between 0930 and 1330 for companionship and social interaction. Visiting the living room can certainly offer help, especially for patients who are a bit confused, says the Stip. “But not too confused.” (Fieldnote 10)*

When the situation does become instable and alternative measures prove futile, while the patient is developing or showing at-risk behavior, becoming a threat to the self or others, the nurses start to consider physical restraints. The nurses collaborate and consider what type of physical restrain is most appropriate in terms of the shown at-risk behavior, the continuation of medical treatment, and the safety of the patient, other patients, and clinical staff. When a physical restraints is chosen, a nurse asks a fellow colleague to assist in the application, keeping an eye on the confused patient and provide extra hands to apply the restraint. To apply a restraint was almost impossibly a sole act. If the at-risk behavior developed gradually upon to the point the confused patient becomes self-dangerous, normally the family or legal representatives as well as the physician are informed about the situation and asked for permission up-front. However, acute situations not always provide the necessary time to do so. Depending on the available staff, a nurse can ask a fellow colleague to call both parties while seeing to the patient, or chose to inform and ask for permission afterward. The effects of the restraint is monitored and reported after application, and reconsidered during handovers and ward rounds.

Dealing with restlessness and confusion was found as a common practice on the ward, used as a way to mitigate the symptoms and prevent the situation from getting worse and restraints from being necessary. However, during the group interview it became clear that not all nurses were aware of the tactics their colleagues used, signaling that lessons could be shared and learned.

### Expect the Unexpected

The nurses underscored the importance of being sensitive toward the situation and rethinking the effectiveness of earlier used solutions as an all-determining way to cope with restless and confused/ at-risk behavior, since actions that worked the day before or a few hours ago might very well be ineffective in the future.

The unpredictability by which restlessness, confusion and at-risk behaviors arise varies widely, not only between patients in general or patients with a comparable clinical picture, but also within patients themselves throughout the day. While this behavior can be anticipated in part based on the clinical intake, the onset can follow a gradually pattern or arise all of the sudden. In the more gradual development, the unpredictability is found in the response of the patient toward the preventive and alternative measures, as finding out what calms a restless or confused patient down is a matter of trial and error, experience with the patient and experience on the ward in general. During an interview, one nurse illustrated this point as a way to anticipate possible incidents:

*Well I think last week, someone who almost fell out of bed and became very restless and was not allowed to be sedated. He had nothing prescribed, so you can’t give him anything. And then you go with the doctor in conclave, like, we can’t go into the night like this. This is going very wrong, he can hurt himself [. . .]then you don’t put the knife to the throat, but I want then that action is being taken, I am with a restless patient [. . .] Eventually, we have given him something, and I sat down with him for a while. Then he calmed down a bit, and at a certain point I found out that he really liked playing football. Then I turned on the TV and then there was peace and quiet. (Interview 11)*

When a situation does become acute, at the time of admission or during hospitalization, the nurses need to deal with the situation instantly, relying on each other’s support to face the situation head on. As a consequence, not only the nurse responsible for the patient but also colleagues need to be prepared to deal with such instances throughout. As such, anticipating what might come, being flexible and looking for creative solutions, in developing situations and instantaneously, is of vital importance when dealing with restless and confused patients.

That a preventive or alternative measure proved helpful earlier, is no guarantee that the same is true later that day or the days to come. Indeed, how well a patient responds to these measures can vary widely, being dependent on the momentarily clinical status, other patients and staff in the vicinity or even the social interaction with the treating nurse. While the effectiveness of said measures can vary between and within patients throughout the day, most nurses on the ward report that at-risk behavior is more likely to arise during evening and night hours. Conversely, the staffing in evening and night hours is lower than during day hours, making the smaller group of nurses more dependent on each other’s skill and knowledge. Knowing how a patient reacted to similar measures during the day is thus important knowledge during night time hours, as well as keeping each other informed during and between shifts.

*During a nighttime observation, a nurse is ringed by a patient, whom with loud music on at 3 a.m., asks for sandwiches, juices and a sleeping pill. The patient has been on the ward for several weeks, and she and her family have shown aggressive behavior toward the nurses and physicians. The nurse is all right with getting the food, but won’t give the sleeping pill. This will make the patient drowsy the next day and that is not good for recovery. In addition, the doctors cannot properly assess her neurological condition in the morning. When all the patients are asleep and everything is cared for, we watch a movie with the other nurses. At the end of the film I suddenly wake up and I appear to have slept for half an hour, just like the nurse. Then we hear from her colleagues that the patient had walked out of her room and went to the emergency door. Her colleague noticed that she was walking out of her room and together with a third nurse they took the patient back to her bed. [The ward is located on the 11* ^th^ *floor of the hospital]. (Fieldnote 20)*

## Discussion

The aim of the study was to study really thoroughly how physical restraints are applied as part of daily routine care of ward nurses on a neurological/neurosurgical ward in a tertiary referral center and how this corresponds and differs from guidelines and protocols. Everyday practice appears to differ significantly from the protocols and work instructions, which were little help to perform good care. We found that the process of applying physical restraints on the ward starts well before restraints are actually used. Initially, the primary concern is to distract or calm a patient down using various preventive and alternative tricks and tactics. As such, the nurses monitor and act upon signs of restlessness and confusion in an early stage, trying to prevent at risk behavior to arise and working together to find an appropriate solution when it does. Indeed, application of restraints was shown to be a shared process in which the nurses collaborated continuously, making use of each other’s experience and skills when deciding on mitigation tactics, choosing which restraint to apply and during the application process in general. Remarkably in the prescribed protocol, work instructions and guideline, little to almost no attention was paid to techniques of dealing with restlessness and confusion, or the importance of experience and mutual collaboration among the nurses. Not surprisingly, the work instructions and protocols were only marginally used by the nurses.

What stands out in the results is how the act of signaling of and dealing with restlessness and confusions is an important precursor of restraint use. That is to say, being keen on signs and hints of restlessness makes the nurses anticipate what might be coming next and respond to it accordingly. While well documented in the domain of mental healthcare (Fernández-Costa et al., 2020; Garriga et al., 2016), attention has been little for this practice in neurological patients although recommendations have been made decades ago (Brower, 1991; Gilbert & Counsell, 1999). As such, restlessness can be seen as a clinical nursing indicator for neurology/neurosurgery ward nurses that a patient can become confused and show at-risk behavior later on. Interestingly, not all nurses seemed to be aware of the diverse preventive and alternative measures used by fellow nurses. Experience played a large part in knowing which tactics were effective in what situations. While this makes indeed hands-on experience on the ward an important factor, sharing effective preventive and alternative measures throughout the team could aid in dealing with restlessness and confusion on the ward appropriately. Furthermore, sharing these lessons could prove valuable insights for other wards within the hospital, as a counterpart for most compliance driven approaches (Maker & McSherry, 2019).

Collaboration and shared experience underscore the importance of team work in dealing with physical restraints. While the nurses indeed showed to have a profound understanding of how to deal with restlessness, confusion and restraints, making use of each other’s experience and judgment was a resource necessary in the use of physical restraints. Communication in this view is vital, as knowing what a colleague knows and can do is of major importance to anticipate how unexpected situations can be handled, what level of advice can be expected, and how well a fellow nurse can aid in monitoring the symptoms of a patient. Having a shared understanding about the level of experience and common practice is thus necessary to know where you stand with each other.

A surprising finding was that the above aspects of physical restraint use on the ward remained almost completely absent in written guidance. Being predominantly concerned with hedging risks in formal work instructions such as obliging formal steps in non-sensical restraint categories, or a heavy reliance on the physicians’ judgment, showed little support for illustrating how dealing with physical restraints can be done right. While this point illustrates the under specification of work descriptions, it also shows the conflict between policy goals and nursing practice. Whereas the guideline was concerned with diligent use of physical restraints and limiting application as much as possible, the context of ward care showed that restraints are often applied with the best interest of the patient in mind. At the same time, the nurses act in the spirit of the protocol, but have to rely much more on practical skills, clinical experience and each other than the protocol and guideline seemed to acknowledge. A sole reliance on the protocol would not result in decreasing the numbers of restraints use any further, nor will a normative inquiry preferring protocol over practice. Appreciatively comparing policy, protocol and practice exposed these conflicts, and offers directions for a wider view on physical restraint application.

**Figure 4.**
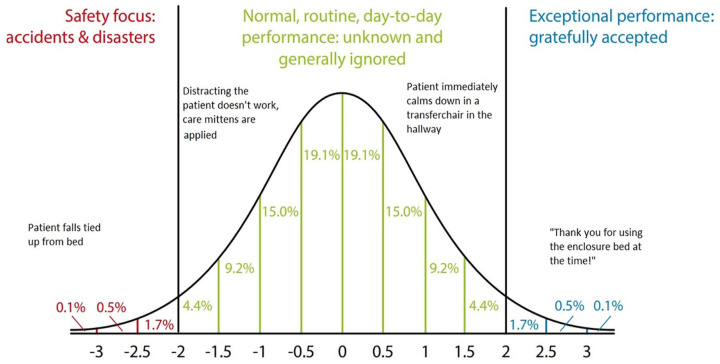
Outline of possible outcomes associated with physical restraint use on the ward. According to Safety-II, outcomes of behavior follow a normal distribution of event probability. While most policy and research efforts focus on preventing incidence on the lefts side, restraining outcomes on the ward had a far wider range possibilities which were far less understood. *Source*. Adopted from Hollnagel, Leonhardt et al. (2013).

One such view could be to regard the incidence of physical restraints as one type of outcome within a larger process of dealing with restlessness and confusion, in which many more outcomes are possible (Figure 4). While the typical focus of guidelines and protocols in restraints use is to prevent bad outcomes from happening on the left side of the curve, our study shows that taking an interest in understanding how all outcomes arise can be very worthwhile. Indeed, many of the tactics and tricks used by the nurses to calm patients down in an early stage, are examples of safe, normal, or even high quality outcomes of dealing with restraints. Increasing the use of mitigating tactics for restlessness and confusion, would also increase instances in which developing at-risk behavior is well managed in an early stage, preventing physical restraints from being necessary, and reduce the number of potential dangerous situations and unwanted outcomes. To arrive at such insights however, an appreciation and in-depth understanding of the structural challenges and surprises met in everyday nursing practice is needed. Taking on the Safety-II perspective as such, our study illustrates how a wider view on physical restraint application can contribute to limiting the incidence of restraint use in the future.

Safe and promising aspects of dealing with physical restraint were already present within the day-to-day activities of the nurses. In the literature, some comparable insights have been found. A recent study by Palese et al. (2021) in over 37 care settings found that a wide range of arguments exist for applying restraints, but that indeed in some instances this is argued for the comfort and independence of the patient. Antonelli (2008) successfully trained nurses in the use of diversion tactics before applying restraints. Ozdemir and Karabulut (2009) found that educational efforts in dealing with agitation on a cardiac ICU helped reduce the amount of applied physical restraints.

However, most of the literature on physical restraints seems predominantly concerned with reduction and prevention of restraint application, leaning on the assumption that all restraint use is an inherent unwanted outcome of everyday clinical nursing (Evans et al., 2003; Kontio et al., 2010; Krüger et al., 2013). Worldwide policies alike seem to follow the same direction (World Health, 2019). Within the hospital setting, often used are training and (re)education efforts to decrease the amount of physical restraints (Abraham et al., 2020), with mixed results. Not surprisingly, as the extent to which restraint use is needed or wanted is highly depended on patient characteristics in the investigated setting, as well as social and cultural forces. Supporting and sustaining what is already there, can provide a welcome alternative to the often top-down implemented quality improvement projects.

Our study highlights possible directions in which occurrence of physical restraints can be reduced or at least their safe use be supported. For the ward under study, encouraging the nursing team to discuss their experiences in being alert for restlessness and mitigating tactics, while sharing their knowledge of preventive and alternative measures, could be a starting point. For wards with frequent occurrence of restless and confused patients, mutual reflection on the difficulties and tricks in mitigating such symptoms could likewise stimulate learning potential. Weighing daily practices with a protocol in this process helps to formulate a common starting point. FRAM stimulated such reflections during the interviews, and could arguably be used as an input for group discussions. Such discussions can help establish a point of reference for the team that is grounded in practice, while also offering room for suggested improvements. While this is not only beneficial for current team members, making explicit current working methods offers new team members insight in their professional development. Another benefit from making explicit current practices is that it could offer outsiders (e.g., managers, policy makers, quality advisors or auditors) valuable lessons in what difficulties are experienced in physical restraint use, and how these can be actively managed and supported.

For other hospital wards where confusion and at-risk behavior are less common, other venues might be more suitable. When the need for physical restraints occurs less frequent on a ward, a straightforward, accessible and easy to use protocol becomes more important for nurses inexperienced with restraint use. Seeking to understand in what places and circumstances such a protocol is used throughout the hospital, questioning if presently available documents are helpful, can surface potential issues early on. Also, utilizing the experience and knowledge of nurses with extensive experience in restraints can provide less-experienced nurses throughout the hospital with quick and in-depth aid. While such routes can be documented in a protocol, building informal networks can also make such expertise better targeted and more accessible when things are getting difficult on less exposed wards.

Arguably enhanced by the frequent occurrence of- and experience with restlessness and confusion with the wards’ inpatients, dealing with restlessness and confusion was found to be a nursing skill vital to the ward nurses’ professional experience with restraint application. In fact, the importance of using shared experience and cooperation in dealing with the unpredictability of appropriate actions to take was underscored as of major importance during the entire process of restraint application on the ward. Presently such aspects of restraint application seemed under appreciated by the organizations’ formal guidance, while conversely being part of everyday clinical nursing on the ward. Failure to make these aspects explicit is a missed opportunity for the ward nurses and safety professionals alike, since elements of safe and deliberate application seemed already present on the ward. Further supporting and maintaining the skillset of dealing with restlessness and confusion throughout the nursing team can provide a valuable direction in stimulating appropriate restraint application on the ward, while offering starting points how to deal with restraints for the larger organization. Placing emphasize on this skillset conversely prevents misguided occurrence of physical restraints, thus offering valuable starting point in supporting patient safety in dealing with these potentially dangerous objects.

### Strengths and Limitations

The strengths of this study can be found in the in-depth ethnographic approach used to thoroughly understand physical restraint use as part of daily nursing activities. As such, the obtained results offer the role of such objects from the viewpoint of the nurses. Furthermore, comparing the usage of restraints against the protocol aided in contextualizing and appreciating the local practices further.

The limitations of the study can be found in the relative difficulty to transfer the exact findings to other contexts in which physical restraints are applied, since practices are likely to vary between national and international neurological wards. Also, the relationship with the formal guidance in the relevant institutions can differ significantly, as Dutch hospitals translate the national guideline toward protocols in line with the hospital specific context.

## Conclusion

Finding a simple solution to restraint use seems a far-fetched goal, and tailor made solutions could be better suited to address this difficult issue. The results of our study highlight the importance of grasping the underlying dynamics behind restraint use in the context of ward care, offering a converse look on adequately understanding, supporting and judging the occurrence of restraint application as a means to sustain and improve patient safety delivered by nurses. To move forward, finding and supporting already safe practices of handling physical restraint provide valuable directions in further decreasing occurrence of physical restraints. The clinical nursing skills and indicators of restlessness and confusion in this study, could be an example of this.

## Supplemental Material

sj-docx-1-gqn-10.1177_23333936221148816 – Supplemental material for Coming to Grips—How Nurses Deal With Restlessness, Confusion, and Physical Restraints on a Neurological/Neurosurgical Ward Click here for additional data file. Supplemental material, sj-docx-1-gqn-10.1177_23333936221148816 for Coming to Grips—How Nurses Deal With Restlessness, Confusion, and Physical Restraints on a Neurological/Neurosurgical Ward by Jaco Tresfon, Kirsten Langeveld, Anja H. Brunsveld-Reinders and Jaap Hamming in Global Qualitative Nursing Research
