# Supplementary material for: Coming to Grips—How Nurses Deal With Restlessness, Confusion, and Physical Restraints on a Neurological/Neurosurgical Ward
Source: Glob Qual Nurs Res. 2023 Jan 24;10:23333936221148816. doi: 10.1177/23333936221148816 (PMC9880574; doi:10.1177/23333936221148816)

**Appendix A: General process.** FRAM of the nurses’ current working methods for applying physical restraints on the ward.


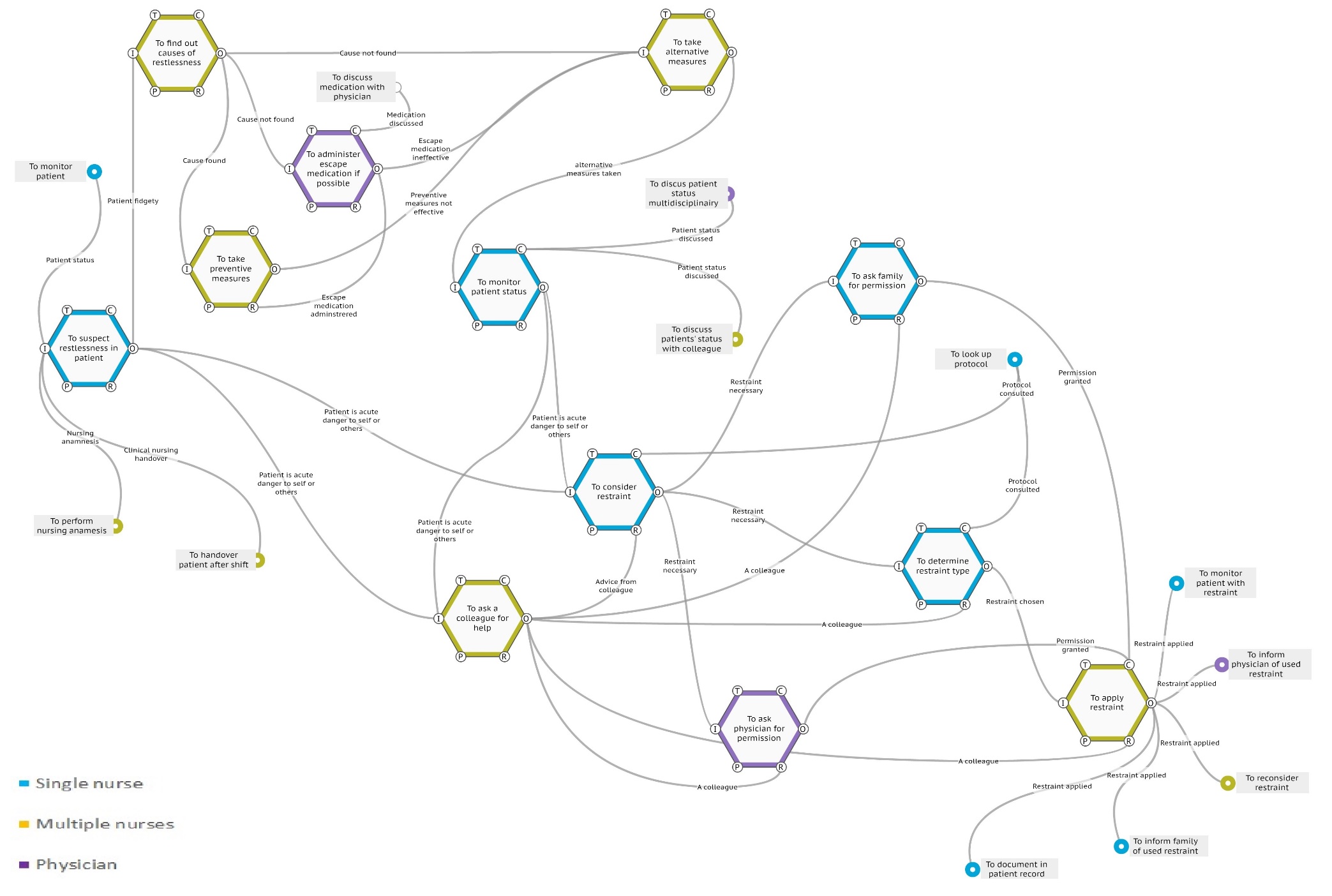

Supplement: sj-docx-1-gqn-10.1177_23333936221148816 – Supplemental material for Coming to Grips—How Nurses Deal With Restlessness, Confusion, and Physical Restraints on a Neurological/Neurosurgical Ward [file sj-docx-1-gqn-10.1177_23333936221148816.docx]
